# An anterograde rabies virus vector for high-resolution large-scale reconstruction of 3D neuron morphology

**DOI:** 10.1007/s00429-014-0730-z

**Published:** 2014-04-11

**Authors:** Matthias Georg Haberl, Silvia Viana da Silva, Jason M. Guest, Melanie Ginger, Alexander Ghanem, Christophe Mulle, Marcel Oberlaender, Karl-Klaus Conzelmann, Andreas Frick

**Affiliations:** 1 Physiopathologie de la plasticité neuronale, INSERM, Neurocentre Magendie, U862, Bordeaux, France; 2 Physiopathologie de la plasticité neuronale, Univ. Bordeaux, Neurocentre Magendie, U862, Bordeaux, France; 3 Institute of NeuroInformatics, University of Zurich, Zurich, Switzerland; 4 Interdisciplinary Institute for Neuroscience, CNRS, UMR 5297, Bordeaux, France; 5 Interdisciplinary Institute for Neuroscience, Univ. Bordeaux, UMR 5297, Bordeaux, France; 6 PDBEB CNC, University of Coimbra, Coimbra, Portugal; 7 Digital Neuroanatomy, Max Planck Florida Institute for Neuroscience, Jupiter, FL USA; 8 Max-von-Pettenkofer Institute and Gene Center of the Ludwig-Maximilians-University Munich, Munich, Germany; 9 Computational Neuroanatomy Group, Max Planck Institute for Biological Cybernetics, Tuebingen, Germany; 10 Bernstein Center for Computational Neuroscience, Tuebingen, Germany

**Keywords:** Neuronal morphology, Connectivity, Sparse labeling, Circuit reconstruction, Neuron-type classification, Alzheimer’s disease

## Abstract

Glycoprotein-deleted rabies virus (RABV ∆G) is a powerful tool for the analysis of neural circuits. Here, we demonstrate the utility of an anterograde RABV ∆G variant for novel neuroanatomical approaches involving either bulk or sparse neuronal populations. This technology exploits the unique features of RABV ∆G vectors, namely autonomous, rapid high-level expression of transgenes, and limited cytotoxicity. Our vector permits the unambiguous long-range and fine-scale tracing of the entire axonal arbor of individual neurons throughout the brain. Notably, this level of labeling can be achieved following infection with a single viral particle. The vector is effective over a range of ages (>14 months) aiding the studies of neurodegenerative disorders or aging, and infects numerous cell types in all brain regions tested. Lastly, it can also be readily combined with retrograde RABV ∆G variants. Together with other modern technologies, this tool provides new possibilities for the investigation of the anatomy and physiology of neural circuits.

## Introduction

The reconstruction of neuronal circuits is central to many questions in neuroscience. Indeed, knowledge of the fine-scale morphology of neurons provides not only insight into the identity and function of individual neurons, but also into the function of neural circuits (Douglas and Martin 2004; Lichtman and Denk 2011; Oberlaender et al. 2012a; Parekh and Ascoli 2013; Svoboda 2011). Successful neuronal reconstruction depends on a number of key parameters: (1) Neurons must be labeled in a way that permits visualization of all neuronal structures (dendrites, spines, axons, boutons). (2) The full extent of neuronal processes should be efficiently labeled. In particular, this applies to the axons, which extend over large brain volumes (Oberlaender et al. 2011). (3) Labeling would ideally permit visualization by high-resolution light microscopy approaches (such as confocal, two-photon, or super-resolution microscopy). In effect, this means efficient expression of a volume-filling fluorescent marker and a high signal-to-noise ratio for the labeled structure. (4) The ideal labeling method would not only be suited to bulk labeling of populations of neurons, but importantly also provide intense labeling of sparse populations of neurons, or even single neurons. This is, because to date the most successful reconstructions of complete neuronal morphology require sparse or single-cell labeling since the axons of bulk labeled neurons become indistinguishable in the densely packed neuropil unless resolved with electron microscopy (da Costa and Martin 2013; Helmstaedter 2013).

Viral vectors fulfill many of these criteria due to their self-amplifying properties (ensuring a high-level expression of volume-filling markers) (Callaway 2008; van den Pol et al. 2009). In particular, genetically modified rabies virus (RABV) is well suited to this approach due to its highly neurotropic nature, rapid, high-level expression of encoded proteins and relatively low cytotoxicity (Wickersham et al. 2007a; Ginger et al. 2013b). The glycoprotein gene-deleted RABV variant, (RABV ∆G) is an especially useful tool that permits the manipulation of the tropism of the virus through pseudotyping approaches (Mebatsion et al. 1997; Ginger et al. 2013b). This principle has previously been exploited for both retrograde labeling of neurons (Wickersham et al. 2007a; Larsen et al. 2007; Nhan and Callaway 2012) and for labeling inputs into a specific cell population (i.e., mono-trans-synaptic tracing) (Wickersham et al. 2007b; Choi et al. 2010).

Here, we employ a RABV ∆G-based method that allows direct transduction of cell bodies, permitting the tracing and complete 3D reconstruction of dendritic and axonal arbors of sparsely labeled neurons. This method combines the advantages of an anterograde tracer with the brilliant morphological labeling previously described for recombinant RABV ∆G (Wickersham et al. 2007a). This vector fulfills all of the aforementioned criteria for neuronal labeling. Low cytotoxicity, fast and strong expression and intense labeling of even the most distant processes set it apart from ‘classical’ viral and non-viral anterograde neuroanatomical tracing approaches. Moreover, the ability to infect a range of cell types over a large age window makes this vector a versatile tool for a large number of experimental situations.

## Results

**Fig. 1:**
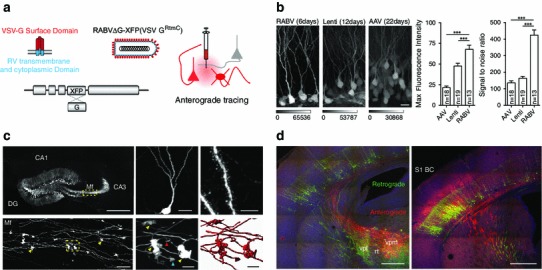
Novel rabies virus variant for anterograde tracing of neuronal morphology. **a** RABV ΔG expressing a fluorescent protein (XFP: eGFP, mCherry or myr-TdTom) was pseudotyped with a chimeric surface protein containing the transmembrane and cytoplasmic domain of the native RABV glycoprotein (RtmC) and surface domain of the G protein of VSV Indiana virus. **b** Comparison of fluorescence intensity and signal-to-noise ratio of cells infected with RABV ΔG (VSV G^RtmC)^ (RABV), VSV G-pseudotyped lentivirus (LV) and adeno-associated virus (AAV). Data are mean ± SEM ***p < 0.001 (one-way ANOVA test). **c** RABV ∆G(VSV G^RtmC^) infection of the hippocampal dentate gyrus (DG) region resulted in intense labeling of granule cells revealing their fine morphological details including the dendritic tree (*upper middle*), spines (*upper right*), mossy fibers (Mf), and mossy fiber boutons (MfBs, *yellow arrowheads*). This permits semi-automated volume reconstruction (*lower middle* and *lower right panel*) of MfBs with their adjacent filopodia (e.g., *red arrowhead*) and satellites (e.g., *blue arrowhead*). *Scale bars* 500 μm, *upper left* 25 μm, *lower left* 15 μm, *upper middle* 5 μm, *upper right*, *lower middle* and *right*. **d** Reciprocal thalamo-cortical and cortico-thalamical projections in S1 BC and VPm following co-injection of retrograde (*green*) and anterograde (red) RABV ∆G. *Scale bars* 500 μm

**Fig. 2:**
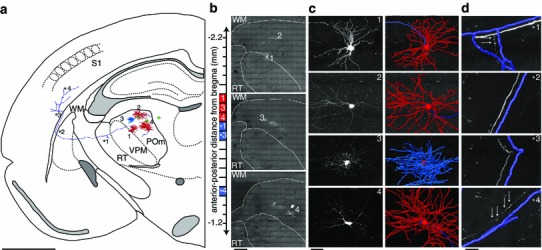
3D reconstruction of thalamic neurons sparsely labeled with RABV ∆G (VSV-G^RtmC^). **a** Three excitatory neurons, one inhibitory interneuron (soma/dendrites: *red*, axon: *blue*) and three astrocytes (*green*) were reconstructed within the imaged volume in thalamus and cortex. **b** Cells were reconstructed from 12 consecutive 50-μm-thick brain sections (*long dashes* of the anterior–posterior axis). Maximum projection images of three sections containing the neuronal somata are shown (*red* within the anterior–posterior axis). **c** *Left* zoom of panel** b**. *Right* Semi-automated reconstructions of the neuronal skeletons are superimposed. In case of the interneuron in* panel 3*, only the soma and axonal arbor are shown. Please note: Reconstructed branches that are not visible in the projection image, such as the gap in the axon in* panel 1*, were traced in adjacent brain sections. **d** One axon was traced into cortex. Maximum projections superimposed with semi-automated reconstructions are shown from exemplary parts of the axon (*blue* within the anterior–posterior axis,* panel* **b**). It branched first within RT (*1), where individual boutons (at *arrow locations*, not reconstructed) indicated potential synapse locations; traversed through the WM (*2, *3), where the axon was perfectly visible; and entered the vibrissal cortex, where labeling quality did not decrease with distance from the soma (i.e., individual boutons were still clearly visible). *Scale bars* 1 mm (**a**) 50 μm (**b**, **c**) and 10 μm (**d**)

**Fig. 3:**
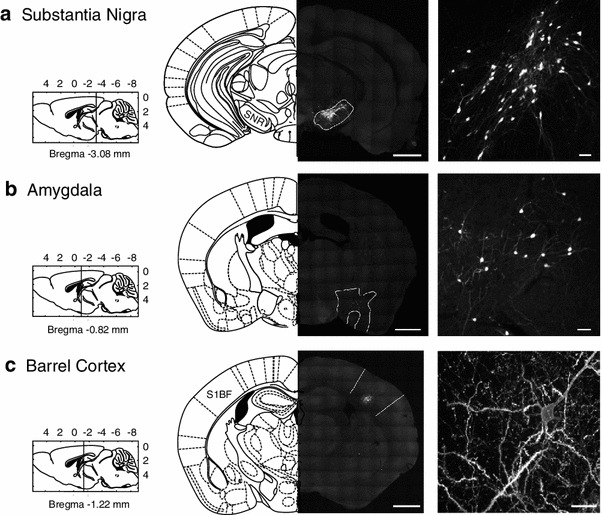
Efficiency of transduction of targeted brain areas. RABV ∆G XFP (VSV-G^RtmC^) transduced cells in all brain structures examined. Confocal microscope images of fluorescently labeled neurons following stereotaxic injection into **a** substantia nigra, **b** anterior amygdaloid, dorsal region, and **c** layers 5 and 6 of the somatosensory cortex. *Scale bars* *left panels* 1 mm, *right panels* 50 μm

**Fig. 4:**
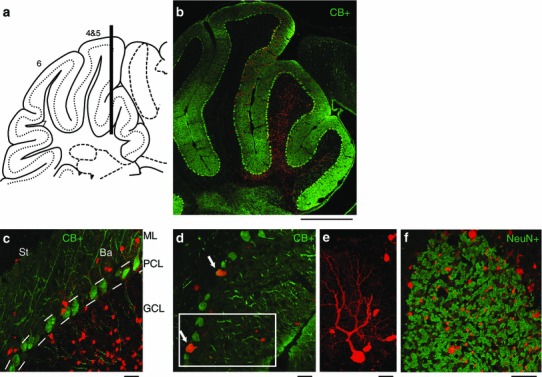
Transduction in the cerebellum. A multiple-step injection at different depths was applied to investigate the transduction efficacy of RABV ∆G (VSV-G^RtmC^) in the cerebellar cortex. **a** Injection scheme, black bar illustrates the virus injections into different depths along the needle track. **b** Injection overview of RABV ∆G (VSV-G^RtmC^) infected cells (*red*) overlaid with calbindin- (CB+) positive cells (*green*). **b**, **c** Transduced cells were found in the molecular layer (ML), the Purkinje cell layer (PCL) and at high abundance in the granule cell layer (GCL). **c** In the molecular layer both of the inhibitory types, stellate cells (St) and basket cells (Ba) were efficiently transduced. Within the Purkinje cell layer (PCL), the calbindin (CB+) immunoreactivity (**d**) and the distinct branching pattern of the dendritic trees (**e**) confirmed the transduction of Purkinje cells. **f** Numerous labeled cells were found in the granule cell layer, where ~80 % of the transduced cells co-labeled for NeuN indicating that the abundant granule cells are transduced efficiently. *Scale bars* 500 μm (**b**) 25 μm (**c**, **d**, **f**) and 10 μm (**e**)

**Fig. 5:**
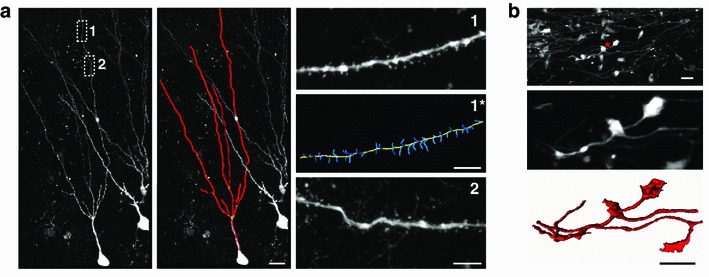
Labeling and surface reconstruction in aged animals. RABV ∆G-mCherry(VSV-G^RtmC^) infection of hippocampal dentate gyrus neurons in a 15-month-old APP/PS1 mouse—a mouse model for Alzheimer’s disease—enables the fine-detailed reconstruction of dendrites and spines (**a**), as well as of axonal boutons (**b**). **a** Strong labeling facilitated automated reconstruction of the dendritic tree (*left* and *middle panel*; scale: 15 μm) and of dendritic spines (*right panels* *1* and 1*; scale 5 μm) of a dentate gyrus cell using Imaris (Bitplane, Zurich, Switzerland). This labeling also revealed anatomical abnormalities like tortuous dendrites, which have previously been described as an effect of aging in humans [*right panel 2* (Tsamis et al. 2010]. **b** Similar to the dendrites/spines, the axons of granule cells (mossy fibers) and their boutons in the CA3 area of the hippocampus were strongly labeled (*upper* and *middle panel*). Automated surface reconstruction (Imaris) of an isolated mossy fiber bouton showing its fine morphological details (*lower panel*). *Scale bars* 5 μm

**Fig. 6:**
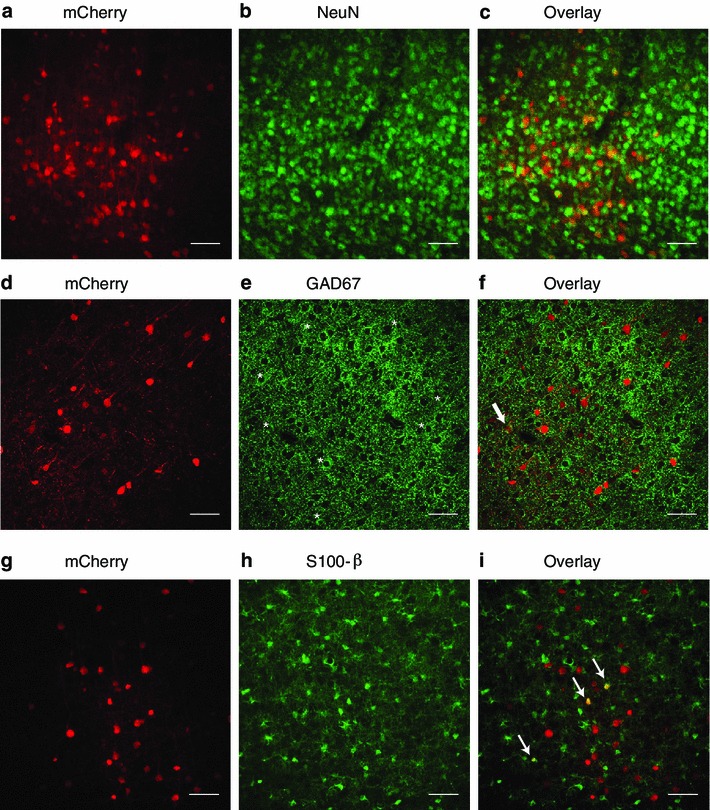
Tropism of RABV ∆G (VSV-G^RtmC^) in CNS. Determination of cell-type identity of RABV ∆G (VSV-G^RtmC^) infected mCherry expressing cells in layer 5 of the barrel cortex (representative images in **a**, **d** and **g**) in 50-μm-thick sections using immunohistochemistry against NeuN (total neuron marker; **b**), GAD67 (marker of inhibitory interneurons; **e**) and S-100β (marker of astrocytes, **h**). Overlay of mCherry and cell-specific marker (**c**, **f** and **i**). Images are either max projections (**a**–**c**, **g**–**i**) derived from selected planes of a multi-plane image stack or a single plane (**d**–**f**) obtained from laser scanning confocal microscopy. *Stars* in panel **e** indicate GAD67 positive cells. A single mCherry-/GAD67 positive neuron is indicated by an *arrow* in (**f**). *Arrows* in panel **i** indicate mCherry-/S-100β positive cells. *Scale bars* 50 μm

To render RABV ∆G capable of cell body infection, we pseudotyped it with a chimeric envelope protein containing the N-terminal domain of the vesicular stomatitis virus glycoprotein (VSV-G). VSV-G binds to highly ubiquitous receptors (Finkelshtein et al. 2013), thus conferring the ability to transduce a wide range of cell types, a property that has previously been exploited for the production of VSV-G pseudotyped viral vectors such as retro- and lentiviruses (Burns et al. 1993). We replaced the membrane anchor and C-terminal cytoplasmic sequence of the authentic VSV-G with that of the RABV-G protein (^RtmC^) to support selective incorporation of the protein into the RABV ∆G envelope (Fig. 1a). We have generated vectors expressing either a membrane-targeted form of tdTomato labeling the neuronal membrane for subsequent surface reconstruction (Fig. 1c, lower panels; Fig. 3c), or cytoplasmic fluorescent proteins (eGFP and mCherry) (all other figures). The resulting vector [RABV ∆G (VSV G^RtmC^)] consistently transduced cells at the site of injection (Figs. 1, 2, 3, 4, 5, 6).

Important criteria determining the suitability of a viral vector for neuronal tracing are the time course for expression, the intensity of expression, and the morphological detail provided by the labeling. Thus, we compared RABV ∆G (VSV G^RtmC^) with two commonly used anterograde viral tracers, namely lentivirus, and adeno-associated virus (AAV). We quantified fluorescence intensity and the signal-to-noise ratio for eGFP expressing neurons following injection of these vectors into the hippocampal dentate gyrus (DG) region (Fig. 1b). Fluorescence intensity and signal-to-noise ratio measured at the cell bodies of transduced neurons were significantly higher for RABV ∆G compared to both other vectors (one-way Anova followed by Tukey multiple-comparison; *p* < 0.001). Importantly, these superior labeling qualities were achieved (with no sign of cytotoxicity) at much shorter time scales of infection (6 days post infection for RABV ∆G (VSV G^RtmC^) versus 12 and 22 days for lentivirus and AAV, respectively). Moreover, dendrites, spines, axons, boutons, as well as filopodia were readily distinguished within RABV ∆G (VSV G^RtmC^) infected neurons, aiding their visualization and reconstruction (Fig. 1c). Notably, the labeled neurons presented here are DG granule cells, neurons that are difficult to infect using the native RABV (reviewed in Ohara et al. 2009).

We noted that the transduction with RABV ∆G (VSV-G^RtmC^) occurred in a purely anterograde manner (i.e., infection of cell bodies and subsequent labeling of axons and dendrites). To exclude the possibility that RABV ∆G (VSV-G^RtmC^) is transported in a retrograde or trans-synaptic manner, we performed injections into the whisker-related barrel cortex (BC) and visually inspected regions known to connect to this structure. While neurons were infected locally at the site of injection, no labeled cell bodies were found in any of the following regions: thalamic ventral posterior medial (VPm) division and posterior medial (POm) division, other areas of the primary somatosensory cortex (S1), secondary somatosensory cortex (S2), primary motor cortex (M1), and contralateral S1 (not shown). In fact, we saw no infected cell outside the local injection site in S1. This property of RABV ∆G (VSV-G^RtmC^) permits its combined use with a retrogradely transducing RABV ∆G variant (Wickersham et al. 2007a) to unambiguously trace projections both to-, and from-, a given region. An example for this type of experiment is shown for the labeling of reciprocal projections between the VPm division of mouse thalamus and the primary somatosensory barrel cortex (S1-BC; Fig. 1d). Importantly, we found a complete absence of retrograde infection in all injections using RABV ∆G (VSV-G^RtmC^).

In the aforementioned examples, we demonstrate the utility of this vector for the transduction of populations of neurons. However, as stated previously, reconstruction of the complete structure of a neuron, including its complex and wide-reaching axonal arbors, requires methods for sparse- or single-neuron labeling. This is often achieved by intracellular filling with biocytin (or its analogs), as most viral-based tracers are completely unsuited to this task. RABV, however, is somewhat unique due to its ability to amplify sufficiently from a single infectious particle (reviewed in Callaway 2008) to confer robust, high intensity labeling to all morphological aspects of an infected cell. Although this property is known from the wild-type CVS strain of RABV (Callaway 2008), it has not been demonstrated for glycoprotein-deleted pseudotyped variants of the SAD B19 strain. To examine the ability of our anterograde RABV ∆G vector to confer the sparse labeling necessary for single neuron reconstruction, we injected 5–10 viral particles into the thalamic POm division of a 16-week-old mouse. Within the investigated thalamic volume of ~400 μm × 400 μm × 400 μm, only seven cells were labeled (Fig. 2a), including three excitatory projecting neurons, one inhibitory interneuron and three astrocytes (Fig. 2a, b). However, all cells were intensely labeled throughout the extent of their processes. Using high-resolution, large-scale confocal microscopy and automated image processing routines (Oberlaender et al. 2007), we reconstructed the somata, dendrites and axons of the four RABV-infected neurons within 12 consecutive 50-μm-thick brain sections (Fig. 2). The high labeling quality in terms of fluorescent intensity, signal-to-noise ratio, and homogeneity across cells (Fig. 2c) including their entire axonal arbor (Fig. 2d) enabled the automated tracing of all morphological fine-structures within the imaged volume. To demonstrate this, we reconstructed the complete 3D morphology of all dendrites, the complete axon of the interneuron, and the initial parts of the axons from the three projecting neurons. Furthermore, we traced and reconstructed one long-range axon. In this case, the neuron projected to the nucleus reticularis (RT) and continued to traverse the white matter tract before entering the vibrissal cortex (S1). This type of axon trajectory has been reported previously for POm neurons (Deschenes et al. 1998). The axon showed no obvious decrease in labeling quality with distance from the soma, allowing identification of individual boutons within thalamus and cortex (Fig. 2d). These results clearly demonstrate the ability of our anterograde RABV ∆G tracer to amplify its genome sufficiently, following infection of a cell by a single particle, to enable tracing of long-ranging axons. As a result of this analysis, we found that the initial axonal segments of the traced neurons were less unidirectional than those described using other methods (Deschenes et al. 1998), possibly indicating that POm projects more diversely to regions other than S1.

To better characterize the efficacy/versatility of our vector, we performed injections into a variety of brain areas and over a range of ages. RABV ∆G (VSV G^RtmC^) transduced cells in all mouse brain areas tested, i.e., somatosensory cortex, thalamus, hippocampal dentate gyrus, substantia nigra and cerebellum (Figs. 1c, d, 2b, 3, 4). RABV ∆G (VSV-G^RtmC^) was capable of transducing excitatory and inhibitory neurons, as well as glial cells, as illustrated in Figs. 2, 4 and 6. For example, in the cerebellum, we found that the virus efficiently transduced inhibitory stellate cells, basket cells and Purkinje cells, as well as the excitatory granule cells. Notably, this vector mediated efficient and intense cell labeling in mice of all ages tested (1–15 months), permitting its use for the neuroanatomical study of neurodegenerative disorders (AD) or aging, as demonstrated in Fig. 5.

To investigate its tropism in the neocortex in greater detail, we performed injections into layer 5 of S1-BC (Fig. 6), where the composition of cell types has been previously well characterized (i.e., ~70 % neurons vs. ~30 % glial cells (Tsai et al. 2009; Meyer et al. 2011)). We found that ~71 % of the labeled cells in layer 5 were neurons (and thus 29 % were glial cells). Interestingly, only 0.4 % of infected neurons were inhibitory interneurons in contrast to the ~80 % excitatory versus ~20 % inhibitory neurons previously reported for layer 5 of the barrel cortex (Meyer et al. 2011). This suggests a strong bias for the transduction of excitatory neurons, as previously reported for VSV-G pseudotyped lentiviral vectors (Nathanson et al. 2009). To further characterize the types of infected glial cells, we used markers for astrocytes [S100β, (Zuo et al. 2004)] and microglia [Iba1, (Schafer et al. 2012)]. We found that ~15 % of infected cells fell into the former category (Fig. 6g–i), while virtually no microglia were labeled (data not shown). We would like to point out, however, that glial cells form a non-homogeneous group of differing origins and several markers co-exist in the various types (Cahoy et al. 2008). Our finding is therefore only the first step in characterizing the infection of various glial cell types by RABV ∆G (VSV G^RtmC^). Nonetheless, our data provide evidence for the ability of pseudotyped RABV ∆G variants to infect glial cells and thus extends the range of cell types that may be amenable to manipulation with RABV ∆G.

## Discussion

Here, we demonstrate the utility of RABV ∆G (VSV-G^RtmC^) for neuroanatomical studies involving not only bulk populations of neurons, but also sparse or individual neurons. Our vector is exclusively anterograde, permits rapid high intensity labeling, without cytotoxic side effects and can be used over a sufficiently extended time window to permit physiological experiments. In addition, it can transduce a range of cell types/brain areas in both young and aged animals. These qualities are unequaled by any other type of viral tracer, and in addition render it useful for the study of aging or neurodegenerative disorders (Fig. 5) where age/toxicity might be a factor limiting labeling success.

We show that RABV ∆G (VSV-G^RtmC^) is able to amplify sufficiently from single-particle infection of a host cell to confer the high intensity labeling necessary for automated detection of morphological features (without prior amplification of the signal) (Fig. 2). Most other viral vectors such as lentivirus and AAV do not provide sufficient labeling intensity for reconstruction upon sparse infection. Indeed, only Sindbis virus (Ghosh et al. 2011) has been reported to amplify sufficiently from a single particle to allow the visualization of long-ranging axonal structures from individual neurons in 3-week-old animals. However, extreme cytotoxicity and reduced efficacy in adult animals (Chen et al. 2000) hamper the practical use of Sindbis virus for the purpose of quantitative morphological tracing.

Our vector may be regarded as a viable alternative to classical single cell labeling approaches, such as those based on biocytin delivery via patch pipettes. The latter are limited by low success rates for recovering complete axonal morphologies [e.g., ~60 % (Oberlaender et al. 2012b)] and require histological post-processing to stain the biocytin-labeled structures. Moreover, reduced penetrability of axon bundles such as the white matter limits the success of tracing long-range axons using biocytin-labeling (where post-processing with immunological agents is required). RABV ∆G (VSV-G^RtmC^), on the other hand, is not affected by any of these issues (Fig. 2). In addition to the aforementioned qualities, the large size of RABV ∆G (VSV-G^RtmC^) particles limits diffusion, allowing very targeted infection of a spatially restricted brain volume. We propose that co-injection of RABV ∆G (VSV-G^RtmC^) variants expressing different fluorescent markers, together with large-scale reconstruction of single-cell morphology, could aid the classification of neuron types within a specific brain region or nucleus.

Recently, a glycoprotein-deleted form of another closely related rhabdovirus, the vesicular-stomatitis-virus (rVSV), has also been used as a single-cycle (i.e., non-trans-synaptic) anterograde tracer (van den Pol et al. 2009). Despite strong morphological labeling following fluorescent marker expression, the use of this virus is limited due to severe, fast-onset cytotoxic effects that cause shut-down of the host cell transcription and nuclear export (Faul et al. 2009). This cytotoxicity strongly restricts the time window for many anatomical/physiological studies to ~1 day post infection, even for attenuated variants of rVSV ∆G (Beier et al. 2011).

The presumed amphotropic qualities of the chimeric VSV-G envelope protein likely enable transduction of a wider range of species and cell types than those presented here. Of note, a similar VSV-G pseudotyped RABV ΔG vector has recently been described (Gomme et al. 2010). This vector was also shown to be anterograde (Wickersham et al. 2013), although it may have a slightly different tropism due to differences in the transmembrane domain of the glycoprotein-packaging construct. Our findings, together with the latter study, suggest novel applications for RABV ∆G in addition to its use as a retrograde (Wickersham et al. 2007a) or mono-trans-synaptic tracer (Wickersham et al. 2007b). For example, RABV ∆G (VSV-G^RtmC^) may be used to define a spatially confined starter cell population for mono-trans-synaptic tracing. It may also be employed as a tool to manipulate/monitor neural circuit activity following the expression of, for example, calcium/voltage indicators or photo-activatable channels (Osakada et al. 2011). In addition, it can be readily combined with retrogradely transducing RABV variants. Unlike other previously reported combinations of anterograde and retrograde agents, the present approach enables the exploitation of two vectors with the same diffusion characteristics, high quality of labeling and short time course for expression as shown in Fig. 1d. Lastly, this tool together with other technologies, e.g., permitting dendritic or synaptic protein profiling (Ginger et al. 2013a; Micheva et al. 2010) or gross-scale reconstruction approaches (as described here), could greatly aid the classification of cell-type identity. In conclusion, the combination of different RABV variants with optical, physiological and computational approaches, offers a wide range of possibilities for the investigation of the structure–function relationship of neuronal circuits.

## Methods

### Engineering of the hybrid glycoprotein

The chimeric VSV/SAD G (VSV G^RtmC^) cDNA was constructed to encode the ectodomain (aa 1–454) of VSV Indiana G (kindly provided by Dr. John K. Rose) fused to the entire transmembrane and cytoplasmic domain (amino acids 450–524) (Mebatsion et al. 1995) of RABV SAD G. This construct differs from the packaging construct employed by Wickersham et al. (2013), which contained the surface- and trans-membrane domain of the VSV glycoprotein and cytoplasmic domain of the RABV glycoprotein.

### Virus production

The production of G-gene deficient RABV SAD ∆G-eGFP and SAD ∆G-mCherry was described previously (Wickersham et al. 2007a). SAD ∆G myr-TdTom was constructed in the same way to encode a protein in which two tandem copies of a myristoylation signal are fused to tdTomato for membrane targeting (Trichas et al. 2008).

Stocks of VSV G^RtmC^-pseudotyped rabies viruses [hereafter referred to as RABV ΔG XFP (VSV G^RtmC^)] were prepared essentially as described in (Rancz et al. 2011), with the exception that BSR T7/5 cells (Buchholz et al. 1999) were used instead of BHK-21 cells and pCAGGS VSV/SAD G was used as the transcomplementing plasmid. Cells were replated 24 h after infection with the initial starter stock and the supernatant media discarded and replaced with new media. VSV G^RtmC^-pseudotyped virus was harvested 3 days post infection. Titers of the different variants of pseudotyped rabies virus were in the range of 2 × 10^6^–5 × 10^7^ infectious particles/ml, estimated by serial dilution and infection of the BHK-21 cell line.

### Stereotaxic injections

All experimental procedures were performed in accordance with French law and the European Directive covering the use of experimental animals (2010/63/EU) and approved by the Ethics Committee of Bordeaux (approval # 5012024-A). Stereotaxic injections were performed as previously described (Cetin et al. 2006) in C57Bl/6 J mice (aged 1–15 months) or in 15-month-old APP/PS1 mice [a mouse model for Alzheimer’s disease (Reiserer et al. 2007)]. In brief, viral vectors were injected into the brains of isoflurane anesthetized and head-fixed mice using a 10-μl glass syringe fitted with a 35-gauge needle or a pulled glass pipette. Volume and speed of the injections were controlled using a WPI Ultra Micro Pump. The stereotaxic coordinates were as follows: (1) Thalamic ventral posteromedial (VPm) nucleus: anterior/posterior (A/P) −1.70 mm, lateral (L) 1.60 mm, dorso/ventral (D/V) 3.20 mm; (2) thalamic posteromedial (POm) nucleus: A/P −1.80 mm, L 1.25 mm, D/V 2.75 mm; (3) hippocampal dentate gyrus (DG): A/P −1.90 mm, L 1.20 mm, D/V 1.90 mm; (4) layer 5 (L5) of primary somatosensory barrel cortex (BC): A/P −0.94 mm, L 3.00 mm, D/V 0.80 mm; (5) cerebellum: A/P −5.68 mm, L 0.68 mm, D/V 1.70–0 mm (6) amygdala: A/P −0.82 mm, L 2.45 mm, D/V 4.75 mm; (7) substantia nigra: A/P −3.00 mm, L 1.56 mm, D/V 4.1 mm. A/P and L coordinates are given with respect to the bregma, D/V coordinates with respect to the brain surface. Injection volumes were 200–600 nl for RABV ΔG XFP (VSV G^RtmC^), 400 nl for AAV (diluted 1/50) and 600 nl for lentivirus. Sparse labeling was achieved by diluting anterograde rabies virus to titers of ~2 × 10^4^ infectious particles/ml and injection of 250 nl, which resulted in injection of 5–10 infectious particles.

### Mouse perfusion and brain sectioning

Mice were deeply anesthetized with a lethal dose of sodium pentobarbital and then transcardially perfused with 30 ml normal Ringer’s solution (135 mM NaCl, 5.4 mM KCl, 1 mM CaCl_2_, 1.8 mM MgCl_2_, 5 mM HEPES, pH 7.4) followed by 100 ml of a 4 % PFA solution (prepared in 1× phosphate buffered saline (PBS, pH 7.4). Fixed brains were dissected and postfixed in 4 % PFA solution for either 24 h, or 6 h in the case where immunohistology was performed. Free-floating slices (50 μm) were cut using a vibratome (Leica).

### Immunohistological determination of the cellular tropism of RABV∆G(VSV-G^RtmC^)

Cell types were identified using antibodies against NeuN (dilution: 1:500; Millipore, clone A60, MAB377) to mark neurons, the 67 kDa isoform of glutamate decarboxylase GAD67 (1:1,500 dilution; Millipore, clone 1G10.2, MAB5406) to label GABAergic neurons (Meyer et al. 2011), S100β (1:1,500; Sigma S2532) to mark astrocytes (Zuo et al. 2004), or against Iba1 (1:500; Wako Cat. #019-19741) to mark microglia (Schafer et al. 2012). NeuN and S100β were detected using Alexa488-conjugated goat anti-mouse H + L (Life technologies), and GAD67 was detected using Alexa647-conjugated goat anti-mouse (subtype IgG2A) (Life technologies).

Immunohistochemical protocols were adapted from Meyer et al. (2011). In brief, free-floating slices were blocked with MOM blocking reagent (Vector Labs) (1 h) in the presence of 0.5 % triton X-100, then for 30 min in 3 % BSA, 4 % NGS, 0.5 % triton in 1× PBS. Slices were incubated 40 h with the primary antibody in 3 % BSA, 2 % NGS in 1× PBS at 4 °C, washed two times with 0.1 M PB then two times with 0.1 M PB + 1 % NGS. Slices were then incubated 2 h at RT with the secondary antibody (1:500) in the presence of 0.3 % triton X-100, washed five times with 0.1 M PB, counter-stained with TO-PRO-3 to label nuclei (1:5,000; Life Technologies), and mounted in prolong gold mounting media. The number of RABV ΔG (VSV G^RtmC^) transduced cells expressing cell-type specific markers was quantified by manually counting the immunohistochemically stained cells in confocal images stacks.

### High-resolution microscopy, tracing and quantification

For reconstruction of thalamic neurons, images were acquired using a prototype confocal laser scanning system (based on LAS AF SP5, Leica Microsystems), equipped with a glycerol immersion objective (HCX PL APO 63x, 1.2 N.A.), a tandem scanning system (Resonance Scanner), spectral detectors with hybrid technology (GaAsP photocathode), and mosaic scanning software [Matrix Screener (beta-version), provided by Frank Sieckmann, Leica Microsystems]. Mosaic image stacks of volumes up to 2 mm × 2 mm × 0.05 mm (in thalamus) and 0.6 × 0.6 × 0.05 mm (in cortex) were acquired at a resolution of 0.094 μm × 0.094 μm × 0.5 μm per voxel (2.5× digital zoom, 8× line average, 8 kHz scanning speed, ~20 × 20 and ~6 × 6 fields of view in thalamus and cortex, respectively) for each of 12 consecutive 50-μm-thick brain sections. 3D reconstructions were performed using previously described automated tracing algorithms (Oberlaender et al. 2007). Automated tracings were proof-edited (Dercksen et al. 2012) and semi-automatically aligned across brain sections (Dercksen et al. 2009) using Amira Visualization Software (Visage Imaging).

All remaining images were acquired using either a commercial confocal microscope (Leica SP5) or a commercial spinning disk system (Leica SP2). Hippocampal mossy fiber boutons were reconstructed from image stacks using the Imaris surface tool (Bitplane, Zurich, Switzerland). To compare the fluorescence intensities and signal-to-noise ratios of RABV ∆G (VSV G^RtmC^), lentivirus- (LV), and adeno-associated virus (AAV) infected brain sections were imaged with identical microscopy settings. Image stacks were acquired at 16 bit-depth at a resolution of 141.47 nm × 141.47 nm × 125.89 nm per voxel (63× magnification, 1.7× digital zoom, 1024 × 1024 pixel per image, 3× line average, 700 Hz scanning speed). Cellular somata were detected automatically in these 3D image stacks using Imaris, and the maximum fluorescence intensity of the somata was quantified as arbitrary unit (0–65536 levels of grey) from 16 bit images. Background levels were calculated for each image stack as the average mean intensity value of several larger distributed areas devoid of any cellular processes (i.e., signal). For illustration purposes, the intensity levels of all three images in Fig. 1b were enhanced to the same extent. Cell counts for immunological cell-type characterizations were performed manually with the use of Amira Visualization Software (Visage Imaging) on confocal image stacks.

### Fluorescence intensity comparison

Fluorescence intensities were compared using brains of mice injected at 4 months of age with either lentivirus (MND-eGFP-WPRE, a kind gift of Dr. N. Abrous), AAV 2/9 CAG eGFP-WPRE (Penn Vector Core), or RABV ΔG-eGFP(VSV G^RtmC^). Injected animals were killed at 6 days post infection (RABV ΔG), 12 days post infection (lentivirus), or 22 days post infection (AAV). eGFP-labeled cells were imaged and analyzed as described above.

### Statistics

Significance was evaluated using one-way ANOVA followed by a post hoc Tukey test for multiple comparisons using GraphPad Prism 6 software (San Diego, CA). *** *p* < 0.001. Data are represented as mean ± SEM.
